# Electrostatic All-Passive Force Clamping of Charged Nanoparticles

**DOI:** 10.1021/acsnano.4c17299

**Published:** 2025-03-04

**Authors:** Yazgan Tuna, Amer Al-Hiyasat, Anna D. Kashkanova, Andreas Dechant, Eric Lutz, Vahid Sandoghdar

**Affiliations:** † Max Planck Institute for the Science of Light, 91058 Erlangen, Germany; ‡ Department of Physics, Friedrich-Alexander University, 91058 Erlangen, Germany; § Department of Physics, Massachusetts Institute of Technology, Cambridge, Massachusetts 02139, United States; ∥ Department of Physics #1, Graduate School of Science, Kyoto University, Kyoto 606-8502, Japan; ⊥ Institute for Theoretical Physics I, University of Stuttgart, 70569 Stuttgart, Germany; # Max-Planck-Zentrum für Physik und Medizin, 91054 Erlangen, Germany

**Keywords:** force clamp, force spectroscopy, electrostatic trapping, gold nanoparticles, nanofluidics, potential mapping, particle tracking

## Abstract

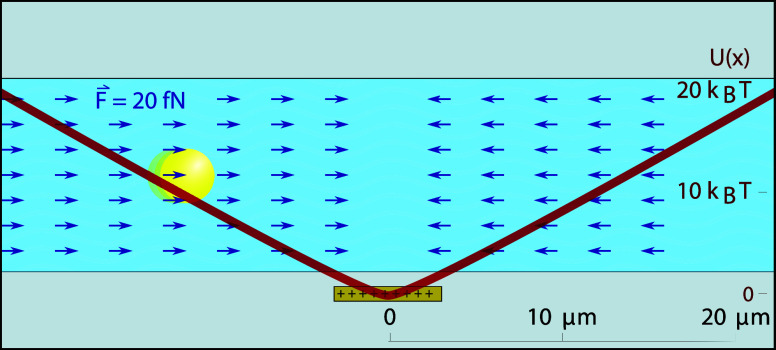

In the past decades, many techniques have been explored for trapping microscopic and nanoscopic objects, but the investigation of nano-objects under arbitrary forces and conditions remains nontrivial. One fundamental case concerns the motion of a particle under a constant force, known as *force clamping*. Here, we employ metallic nanoribbons embedded in a glass substrate in a capacitor configuration to generate a constant electric field on a charged nanoparticle in a water-filled glass nanochannel. We estimate the force fields from Brownian trajectories over several micrometers and confirm the constant behavior of the forces both numerically and experimentally. Furthermore, we manipulate the diffusion and relaxation times of the nanoparticles by tuning the charge density on the electrode. Our highly compact and controllable setting allows for the trapping and force-clamping of charged nanoparticles in a solution, providing a platform for investigating nanoscopic diffusion phenomena.

Many biological and biophysical studies aim to understand and quantify the application of forces.^1−4^ Active generation of forces in the intracellular environment by molecular motors,^4−6^ actin and tubulin polymerization,^4,7^ DNA-cytoskeletal attachments,^8^ and covalent bonding,^9^ typically lie in the piconewton (pN) range. Entropic forces that can affect the function of biological structures such as mechanical susceptibilities of biointerfacial structures,^9^ formation of DNA loops,^8,10^ and extension of single DNA molecules^11^ are in the order of several tens of femtonewton (fN). To investigate these processes, it is helpful to apply external forces of comparable size, for example, by using optical tweezers (0.1–100 pN)^12,13^ and magnetic tweezers (down to 10 fN).^14−17^ In particular, many studies on molecular motors or unfolding proteins would benefit from holding the nano-object at a constant force, a procedure known as *force clamping*.^18−20^

The most well-established technique for force clamping nanoparticles is to use an optical trap coupled to an electronic feedback system that tracks the instantaneous particle position and adjusts the trap to maintain a constant force.^12,13^ This and other active trapping methods, such as Anti-Brownian Electrokinetic (ABEL) trapping^21,22^ and optoelectronic tweezers^23,24^ are inherently limited by the finite bandwidth of the feedback loop or the response time of the digital micromirror devices, typically less than 1 kHz.^25^ Several biological processes of interest, however, such as dwell times of molecular motors,^4,26^ folding/unfolding,^27,28^ and diffusion of proteins,^29,30^ entail dynamics at submillisecond time scales.

Passive force clamping overcomes temporal limitations by applying a position-independent force field to the probe particle. Here, one usually chooses a regime, where the potential applied by a magnetic tweezer^14−17^ or an optical force^12,13^ can be approximated as a linear potential.^31^ However, this approach is often difficult to implement and is limited in scope. For instance, magnetic tweezers necessitate the use of particles that possess a sufficiently large magnetic permeability for manipulation, which restricts the method to nanoparticles with size in the order of 100 nm or larger. In addition, permanent magnets have to be situated and displaced close to the sample, imposing geometric constraints on the experimental setup and restricting the response time.^14−17^ Passive all-optical force clamping, on the other hand, can be fairly fast but it has a strongly restricted range, typically less than 1 μm.^31^

## Results and Discussion

In this work, we introduce a controllable experimental scheme that can trap and passively force-clamp charged nanoparticles in an aqueous solution. We develop an apparatus that generates a linear electrostatic potential over a region of about 5 μm. The magnitude of this potential can be rapidly and precisely tuned within a range of a few fN with temporal resolution on the order of tens of microseconds. The temporal resolution is determined by the charge relaxation time of the electrolyte, while the precision of force detection is limited by the length of the particle trajectory (Supporting Information). This capability enables the application of well-defined forces to probe particles at the biological time scales.^32,33^ We present a procedure for calibrating our passive force clamp using the statistics of Brownian trajectories of gold nanoparticles (GNP).

**Figure 1:**
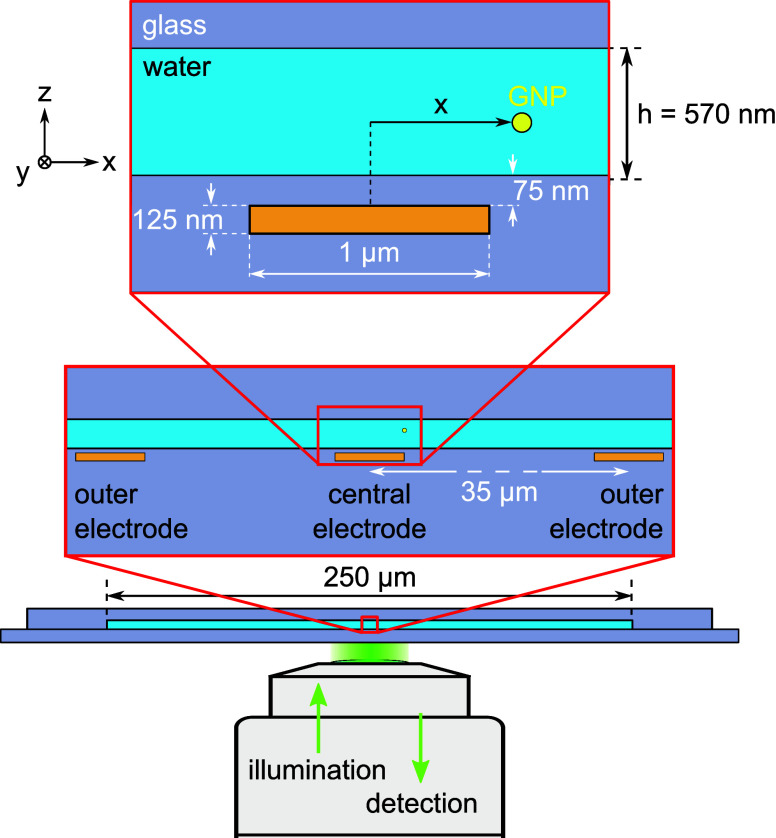
Schematics of the experimental setup. Three ITO electrodes are embedded in a silica substrate. A second silica substrate is bonded on top to form a nanochannel of height *h* over a length of 7 mm in the *y* direction. A gold nanoparticle (GNP) of 80 nm in diameter diffuses in water inside this nanochannel. A microscope objective is used to image and monitor the GNP via wide-field iSCAT microscopy. Various relevant dimensions and information are presented in two nested close-ups.

The most straightforward approach to creating a linear electrostatic potential is to place two charged plates in a parallel arrangement^34^ which has been the most commonly used scheme for the electrophoretic manipulation of particles.^35,36^ However, this configuration would push a charged particle toward the oppositely charged plate, severely limiting the observation time and making force calibration and statistical studies challenging. To get around this difficulty, we created an attractive linear potential established between three parallel conductive nanoribbons in a plane. As illustrated in Figure 1, structures of thickness 125 nm, width 1 μm, and length 7 mm, were embedded in a fused silica substrate at separations of 35 μm. The resulting substrate was then bonded to a fused silica substrate with suitable indentation to create a nanochannel of a few hundred nanometers in height (Supporting Information). Capillary forces were exploited to introduce an aqueous suspension of GNPs with a diameter 80 nm into the nanochannel. The line charge densities (λ) on each nanoribbon electrode and thus the magnitude of the electrostatic potential can be controlled via externally applied voltages between the central (positive) and outer electrodes (negative).

**Figure 2:**
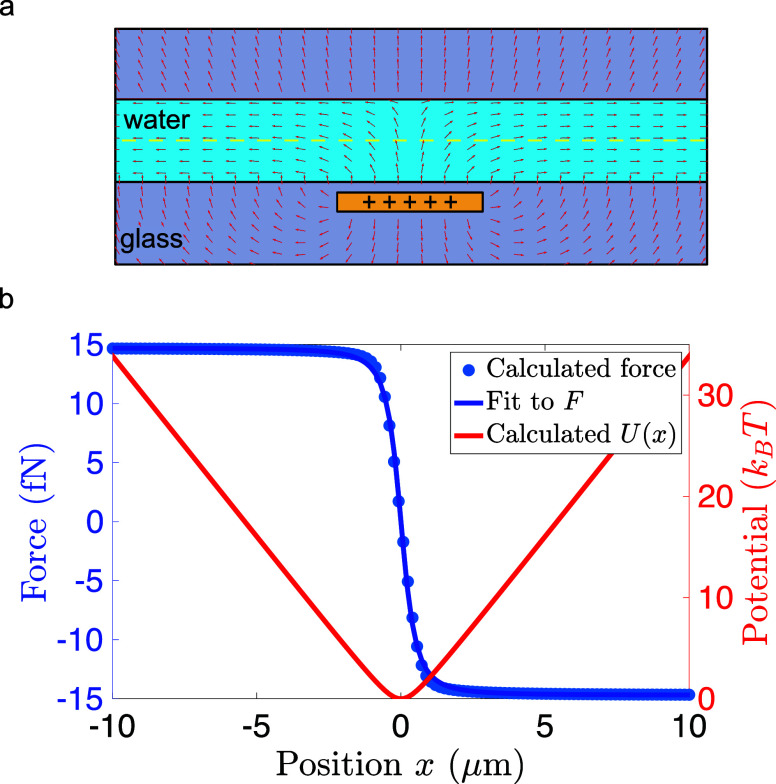
Electrostatic force fields and potentials. (a) Force field generated by a charged 3-electrode capacitor (see Figure 1) on a negatively charged particle (100 *e*^–^). Arrowheads show the direction of the force. The length of the arrows is normalized. (b) Electrostatic force (blue) and potential (red) as a function of the lateral displacement *x* evaluated at the midplane of the channel (see dashed line in (a)).

Textbook expectation of the electric potential surrounding a charged wire is a logarithmically decreasing function of the radial distance from the wire.^34^ However, the large difference of the electric conductivity at the interface of fused silica (σ_sil_ = 1 × 10^–10^ S/m) and water (σ_wat_ = 5.5 × 10^–6^ S/m) leads to a substantially different functional form of the potential (*U*(*x*)) in the nanochannel. We used numerical simulations to determine *U*(*x*) by solving Maxwell’s equations in COMSOL Multiphysics 6.0.^37^ Here, we considered our experimental conditions of an electrically charged indium tin oxide (ITO) ribbon embedded in isolating glass under a nanochannel filled with deionized water (see Figure 1). The predicted force field is depicted in Figure 2a, and the resulting potential is well approximated by
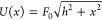
1where *x* denotes the position of the nanoparticle in the substrate plane along the axis perpendicular to the electrodes (see Figure 2b). For large distances, the potential (red) approaches a linear function, *U*(*x*) = *F*_0_|*x*|, where *F*_0_ represents the constant force (blue) acting on the particle determined by the particle charge and the voltage through the wire. We find that the water–silica interfaces at the upper and lower channel walls serve to squeeze the electric field lines toward constancy within the channel volume, providing an electrostatic force clamp in the region |*x*| ≳ *h*.

The motion of a charged nanoparticle along the *x* direction is well described by an overdamped Langevin equation,
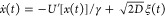
2for Brownian motion^38^ in an external potential *U*(*x*), where *D* is the diffusion coefficient, γ denotes the damping constant, and ξ(*t*) is an uncorrelated Gaussian white noise force with unit variance. The dynamics of the particles can be tuned by varying the charge on the wire.

**Figure 3:**
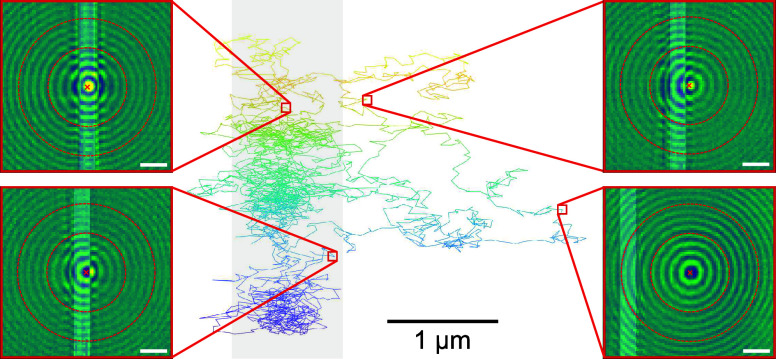
Portion of a trajectory and distorted point spread functions. A 3 s section of a 48.6 s long trajectory is overlaid on the position of the nanoribbon (gray shaded area). Colors encode the temporal evolution of the trajectory: blue marks its beginning and yellow marks its end. Representative interferometric point spread functions (iPSF) after background subtraction are depicted in the red boxes on the sides. The central parts of the iPSFs are somewhat distorted due to scattering from the electrode, while the outer rings remain mostly unperturbed. The dashed red circles represent the region where the rings are used for particle localization. Scale bars indicate 1 μm.

To image the motion of individual GNPs near a charged electrode, we employed wide-field interferometric scattering microscopy (iSCAT).^39,40^ In this technique, the light scattered by a nanostructure interferes with a reference light, which in our case is provided by the reflection from the bottom of the sample chamber. The interferometric point spread function (iPSF) in the wide-field mode consists of a central lobe and many concentric rings.^41^ In our case, the image of a nanoparticle is perturbed by the scattering from the electrode when their positions overlap. However, as displayed in Figure 3, the outer rings remain preserved. We can, therefore, determine the position of a GNP with a localization error of less than 10 nm (around 0.07 px) (Supporting Information) by employing an algorithm based on the radial symmetry of the outer rings.^42^ The localizations of a particle in 48,681 frames (only 3000 frames are shown in Figure 3) were then combined to generate a trajectory.

To verify the predicted constant force field, we used a statistical force estimator that maps arbitrary potentials from measured Brownian trajectories.^43−45^ This estimator approximates the force at each position *x* by calculating the average drift of the particle steps that begin within a small neighborhood of *x* (Methods). For the case of the experiment, this estimation scheme is equivalent to more involved techniques such as Bayesian inference methods.^46,47^ We estimate the force constant *F*_0_ for each single trajectory by fitting the function
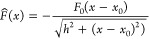
3to the estimated force. Since the force depends on the known wire voltage and the unknown (different for every particle) charge of the nanoparticle, we introduce rescaled variables, defined as *x̃* = (*x* – *x*_0_)/*h* and *F̃*(*x̃*) = *F̂*/*F*_0_, where *x*_0_ is an arbitrary shift. We can accordingly rewrite the estimated force in the dimensionless form , which allows us to combine the data from different particles onto a common scale.

**Figure 4:**
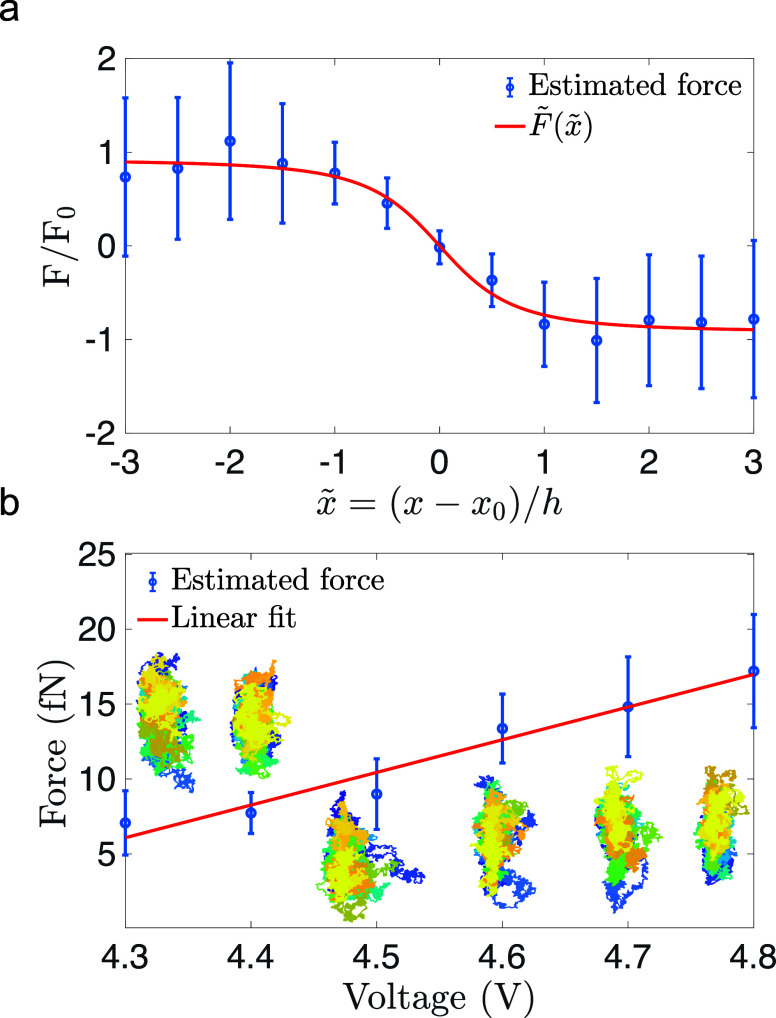
Measured force field and tunability. (a) Blue circles show the estimated force from 107 rescaled experimental trajectories. The solid red line is a fit according to eq 3. We find that the force becomes constant for |*x*| ≳ 1 μm. (b) Linear dependence of the force (blue circles) on the applied voltage for the trajectory of a single particle depicted in each inset. Colors encode the temporal evolution of the trajectory: blue marks its beginning and yellow marks its end. Error bars correspond to one standard deviation. The red line indicates a linear fit function.

Figure 4a displays the estimated dimensionless force field obtained by averaging over 107 rescaled trajectories. The solid red line shows the rescaled function *F̃*(*x̃*). The very good agreement with the experimental data supports the assumption that the force is constant far from the central electrode (for |*x*|≳ *h*). We emphasize that the parameters *F*_0_, *x*_0_ and *h* can be determined for each individual trajectory and not just for the averaged dynamics. The magnitude of the force can be tuned by modulating the wire voltage. Figure 4b shows the linear change of the force on the trajectory of a single GNP (presented in the inset) as the wire voltage is varied from 4.3 to 4.8 V. Our findings confirm that the setup applies a constant force toward the wire over a region of a few micrometers.

**Figure 5:**
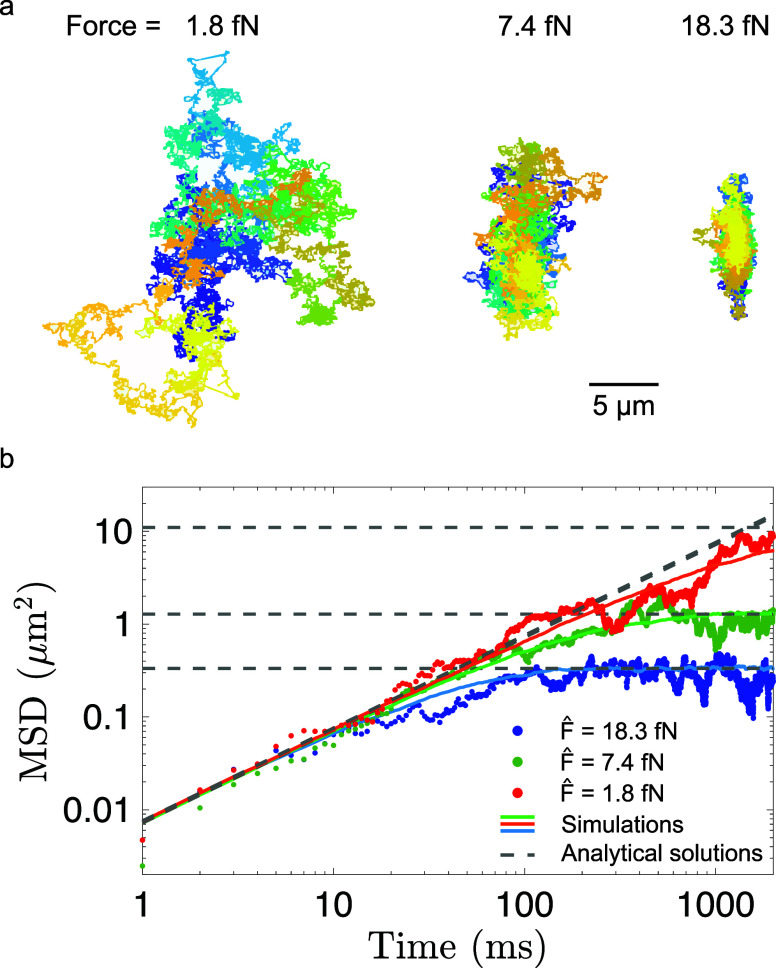
Mean squared displacement. (a) Three exemplary experimental trajectories for estimated weak (*F̂* = 1.8 fN), moderate (*F̂* = 7.4 fN) and strong (*F̂* = 18.3 fN) forces. Each trajectory was 50 s long. Blue and yellow encode the beginning and the end of the trajectory, respectively. (b) Measured mean square displacements (MSD) for the corresponding trajectories (dots) are compared against the numerical (solid lines) and asymptotic analytical predictions (dashed gray lines). Relaxation times decrease with increasing force.

We additionally tested the consistency of the above analysis by investigating the diffusion properties of the nanoparticles. To achieve this, we compared the experimentally determined mean squared displacement (MSD) for select trajectories (*F*_0_ = 1.8 fN (red), *F*_0_ = 7.4 fN (green), *F*_0_ = 18.3 fN (blue)) to the one expected in the estimated force field (see Figure 5). We computed the MSD numerically since the nonlinear dependence of the force (see eq 3) does not permit an analytic expression at arbitrary times. We observe free diffusion at short times with a linear time dependence and an average diffusion coefficient of *D* = 3.8 μm^2^/s, as well as thermalization inside the confining potential at larger times after which the MSD is time independent (see Figure 5b). The thermalization time, ranging from a few hundred to a few thousand milliseconds, depends on the value of the force and can be controlled via the voltage applied to the electrodes. We obtain good agreement between the experimental data (dots) and numerical results (solid lines), as well as with asymptotic analytical approximations (dashed gray lines) that are valid for free diffusion at short times and diffusion in the force field (see eq 3) at large times (Supporting Information).

## Conclusions

In conclusion, we have developed a passive electrostatic force clamp that works on all time scales for charged nanoparticles. The electrostatic force clamp presented in this work operates in an aqueous solution and can be used to apply forces from a few fN to several pN. The relatively long-range of the linear potential is well suited to biophysical experiments where micron-sized displacements are expected. Moreover, its compatibility with other imaging techniques, such as fluorescence microscopy and phase microscopy, as demonstrated in this study, enhances its versatility. In particular, the combination of our device with the high speed of iSCAT microscopy in the hundreds of kHz^30,48,49^ and time-varying voltages applied to the electrodes opens doors to interesting investigations such as the selective trapping of particles,^50^ dynamic manipulation of proteins and nucleic acid assemblies.^33,51−53^

Additionally, our device facilitates electrostatic trapping near electrodes. The linear potential offers a broader trapping range and higher sensitivity when adjusting the potential depth compared to quadratic potentials. Furthermore, our microfluidic scheme has the advantage of on-demand single-molecule trapping in solutions when compared to geometry-induced electrostatic trapping schemes.^54,55^ Our experimental scheme can also be operated with an A.C. field for sorting and valving of nanoscopic entities,^56^ electrophoretic trapping of nanoparticles and macromolecules,^57,58^ in microfluidic applications. Moreover, the geometric scalability of the electrostatic potential in our platform enables the use of dielectric particles at the micron scale in channels with suitable heights (see Supporting Information), offering opportunities such as for studying probe interactions with cells,^59,60^ membrane diffusion,^61,62^ and electrophoretic manipulation of cells and particles.^63^ Collectively, these capabilities establish our experimental approach as a versatile tool for diverse studies in nonequilibrium statistical mechanics.^64^

## Methods

### Device Fabrication

A fused silica substrate (thickness 170 μm) is structured using photolithography patterning and buffered-oxide (BOE) wet etching. 125 nm indium tin oxide (ITO) and 75 nm SiO_2_ are sputtered on the etched substrate (200 nm), and the lift-off process is performed in acetone via ultrasonic treatment. The nanochannels are patterned on a thick (1 mm) fused silica substrate using photolithography and etched 570 nm using reactive ion etching.

The nanochannel and structured substrate are cleaned twice, using acetone, isopropanol, and 50% Extran solution in order. After the plasma treatment on both surfaces, the nanochannel structured substrate is bonded to the wire-structured substrate via van der Waals forces. Then the sample is kept at 750 °C for at least 24 h to reach irreversible glass–glass covalent bonding.

### Measurement

The GNP solution (80 nm, Sigma-Aldrich) is prepared and added from one side of the channel entrance, and the suspension is sucked inside via capillary forces. Once the channel is filled, another droplet of the same suspension is added to the other end to equalize the pressure and eliminate the drift. After several minutes of stabilization, the steady state is reached and a voltage is applied to create the desired potential shape and depth across three parallel electrodes (Supporting Information). The sample is illuminated from the bottom with a 532 nm laser and a standard wide-field iSCAT measurement is performed at a 1 kHz frame rate (Supporting Information). The motion of particles is recorded for the longest possible time period which is limited by the field of view of the microscope. The measurement is repeated at different voltages but not necessarily with the same particle or at the same spatial position.

### Image Processing and Trajectory Extraction

First, we eliminate the static features from each frame of a video using aligned median background subtraction. We note that due to the 3-wave interference (light scattered by the GNP, light scattered by the wire, and light reflected from the sample), the PSF of the particle is distorted in the vicinity of the wire. Therefore, we localize the GNP in each frame by fitting a Gaussian to the center of radial symmetry of the higher-order interference rings,^42^ which are not distorted (Supporting Information). The individual localizations are then linked to form a trajectory using the trackpy package.^65^

### Force Estimation

To estimate the shape of a generic potential by mapping the force field that a Brownian particle experiences,^43−45^ we define a region of width *l* around each position *x*, *I*(*x*) = (*x* – *l*/2, *x* + *l*/2), such that *U*(*x*) is roughly constant within *I*(*x*). Therefore, steps that begin within the interval *I*(*x*) can be pooled together and averaged to calculate step size distribution which reduces to a Brownian motion with constant drift at short times. The estimator for the force (*F̂*), *U′*(*x*) accordingly becomes , where *v*_*i*_ is the size of the *i*th step.

## Supporting Information Available

Fabrication details of the nanofluidic device; experimental measurement procedure; effects of electroosmotic flow, gravitational forces, and ionic strength; calculation of the electric fields; details and the characterization of the image analysis; estimation of forces from Brownian trajectories; and analytical solution to the MSD for linear potentials (PDF)
