# Supplementary material for: Electrostatic All-Passive Force Clamping of Charged Nanoparticles
Source: ACS Nano. 2025 Mar 4;19(10):10173–9. doi: 10.1021/acsnano.4c17299 (PMC11924585; doi:10.1021/acsnano.4c17299)
Supplement: Supplementary file 1 — nn4c17299_si_001.pdf [file nn4c17299_si_001.pdf]

# Electrostatic all-passive force clamping of charged nanoparticles

Yazgan Tuna,[1,2] Amer Al-Hiyasat,[3] Anna D. Kashkanova,[1] Andreas Dechant,[4] Eric Lutz,[5] and Vahid Sandoghdar[1,2,6]

[1] *Max Planck Institute for the Science of Light, 91058 Erlangen, Germany*
[2] *Department of Physics, Friedrich-Alexander University, 91058 Erlangen, Germany*
[3] *Department of Physics, Massachusetts Institute of Technology, Cambridge, MA 02139, USA*
[4] *Department of Physics #1, Graduate School of Science, Kyoto University, Kyoto 606-8502, Japan*
[5] *Institute for Theoretical Physics I, University of Stuttgart, 70569 Stuttgart, Germany*
[6] *Max-Planck-Zentrum für Physik und Medizin, 91054 Erlangen, Germany**

## I. SAMPLE PREPARATION

We design 3 parallel electrodes, each 7 mm long, and 1 μm wide in the measurement area. This establishes a capacitor configuration to allow fine-tuning of the line charge density (λ) via an applied voltage. The electrodes are fabricated from indium tin oxide (ITO) due to its suitable optical properties such as a refractive index of 1.93 (at a wavelength of 532 nm) and transparency of about 80% for a 125 nm thick film at a glass interface. Another important feature of ITO is its high-temperature tolerance (∼ 800 °C), which is crucial for the high-temperature glass-glass bonding process required at the later stages of fabrication.

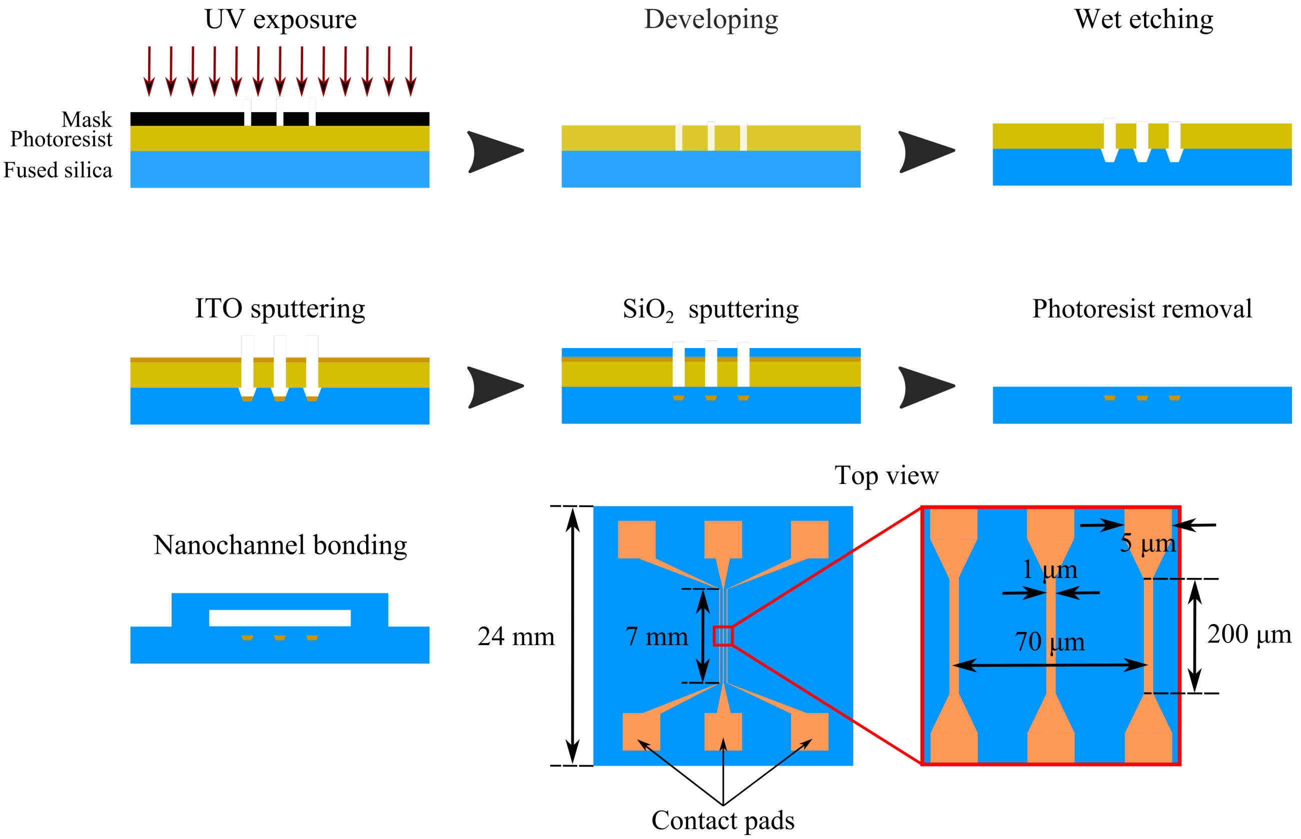


FIG. 1. The fabrication process. A positive photoresist is coated on fused silica and structured via UV exposure. The substrate is then wet etched with buffered oxide etchant (BOE) about 200 nm, and the etched wells are filled with, first ITO and then $SiO_2$ via sputtering. Next, the photoresist is removed. Finally, the surroundings of the nanoelectrodes are enclosed by bonding an indented fused silica substrate to create a nanochannel.

* vahid.sandoghdar@mpl.mpg.de

We first spin-coat a positive photoresist (AZ MIR701) on a pre-cleaned fused silica substrate. Next, we structure it via UV exposure (SUSS MicroTec, mask aligner MA/BA6) through a chromium mask. Then, after developing the sample (AZ 726mif), the substrate is wet-etched by about 200 nm with 4% buffered oxide etchant (BOE). Then, ITO is sputtered in 2 steps to reach a thickness of 125 nm, followed by 75 nm sputtering (AJA International, Inc. ATC Orion sputtering system) of $SiO_2$ in order to isolate the electrodes from the environment. The lift-off process is performed via acetone in an ultrasonic bath until all resist residues are removed.

The width of the electrodes is 5 µm at the two ends and is thinned to 1 µm within the central measurement area. The total length of the electrodes is 7 mm. The electrodes are enlarged to resemble large contact pads where the voltage is applied. The final structure has 3 parallel electrodes with a separation of 35 µm from each other (See Fig. 1).

For the nanochannel formation, another fused silica substrate (10 mm × 10 mm) is dry-etched (Oxford Instruments, The Oxford Plasmalab 100 Cluster) 570 nm deep over the length of 10 mm and the width of 250 µm via the same photolithography process. Before the formation of the nanochannel on the nanoelectrode structured substrate, we clean and functionalize both surfaces by means of plasma treatment. The nanochannel substrate is attached on top of the nanoelectrode structured substrate via van-der-Waals interactions with the assistance of methanol. After the methanol is completely evaporated under ambient conditions ($\sim$ 3 hours), the sample is heated to 750 °C for at least 24 hours in order to create irreversible covalent bonding. After the sample is slowly cooled down, it is ready for measurement.

## II. MEASUREMENT PROCEDURE

We use gold nanoparticles (GNPs, Sigma Aldrich) with a diameter of 80 nm due to their large amount of surface charge ($\sim 100\ e^-$) [1], which is important for interaction with the charged electrodes. Moreover, their relatively large size results in a high signal-to-noise ratio for tracking. Yet, they are small enough so their motion is dominated by thermal noise. The GNP suspension with a pH of 5.9 and an ionic concentration of about 1.2 µM is added from one side of the channel and capillary forces suck the suspension inside the nanochannel. Once the channel is filled, another droplet of the same suspension is added to the other end to equalize the pressure and eliminate the drift. After several minutes of stabilization, the steady state is reached and a voltage is applied among three electrodes. The central electrode is charged positively while both ends are negatively charged. We record the x-y plane projection of GNP's motion and its motion in the z-plane is minimized down to a level where it is negligible compared to the trapping length scale ($\sim 2-3$ µm) by nanochannel configuration. The glass nanochannel walls induce an electrostatic force within the Debye length-scale ($\sim$ 270 nm) and therefore keep charged GNPs away from the surfaces [2].

We employ interferometric scattering microscopy (iSCAT)[3, 4] (See Fig. 5a) which allows us to track small particles with high precision at high frame rates. The motion of the GNPs is recorded at 1 kHz for the longest possible time period which is limited by the field of view of our microscope. The measurement is repeated at different voltages but not necessarily with the same particle or at the same spatial position.

## III. THE EFFECTS OF ELECTROOSMOTIC FLOW, GRAVITATIONAL FORCES, AND IONIC STRENGTH

Our electrostatic force-clamping scheme relies on the induction of a constant DC field within the nanofluidic channel. Consequently, estimating the contributions of electroosmotic flow and gravitational forces to the measured forces is important. The electroosmotic mobility can be calculated as:

$$\mu_{EO} = \frac{\epsilon\zeta}{\eta}, \tag{1}$$

where $\mu_{EO}$ is the electroosmotic mobility, $\epsilon = \epsilon_r \epsilon_0$ is the permittivity of water, $\zeta$ is the zeta potential, and $\eta$ is the dynamic viscosity of water. The zeta potential of the fused silica slits has been studied by Gerspach et al. [5], who estimated the apparent zeta potential approximately 250 nm away from the surface to be around 0 to -1 mV.

Assuming $\zeta = -1$ mV, $\eta = 0.001$ Pa·s and $\epsilon = 70.8 \times 10^{-11}$, the electroosmotic mobility is calculated as

$$\mu_{EO} \simeq 7 \times 10^{-10}\ \mathrm{m^2/(V\cdot s)}. \tag{2}$$

The electroosmotic velocity is then given by:

$$v_{EO} = \mu_{EO} \times E = 7 \times 10^{-7} \text{ m/s} \tag{3}$$

where the electric field strength is  1000 V/m corresponding to a 5 V potential difference (less than the maximum experimentally applied voltage) between the central and outer electrodes (see Figure 2a). The electroosmotic drag force acting on an 80 nm particle is calculated as

$$F_{EO\ drag} = 6\pi\eta r v_{EO} = 0.5 \text{ fN}. \tag{4}$$

Gravitational forces also contribute to the system, albeit minimally. Using the density of gold ($\rho_{\text{gold}} = 19320$ kg/m$^3$), the density of water ($\rho_{\text{water}} = 1000$ kg/m$^3$), and gravitational acceleration ($g = 9.8$ m/s$^2$), the gravitational force on an 80 nm particle is calculated as:

$$F_g = V\Delta\rho g = 2.68 \times 10^{-22} \times 18320 \times 9.8 \simeq 5 \times 10^{-17} = \ 0.05 \text{ fN} \tag{5}$$

Lastly, we estimate the impact of ionic strength and surface charge screening on the force clamping of charged nanoparticles. Gold nanoparticles carry a net negative surface charge density of $\sigma = 0.8 \times 10^{-4}$ C/m$^2$, resulting in a zeta potential of approximately -35 to -40 mV in ultrapure water [5, 6]. As the electrolyte's salt concentration increases, surface charges are screened, leading to a reduction in zeta potential. For instance, Habib et al. [7] reported a decrease in the zeta potential of GNPs from -26.56 mV to -14.92 mV when the concentration of 2-morpholinoethanesulfonic acid (MES) at pH 6 increased from 1 mM to 100 mM. Similarly, Wang et al. [6] observed that surface modifications, such as coating GNPs with citrate-, thiolated single-stranded DNA, or proteins, significantly enhance the zeta potential. They measured zeta potential of -13.3 mV (compared to ≃40 mV in pure water) at 300 mM NaCl, where particles started to aggregate. Therefore, although physiological salinity levels might affect the performance of our force-clamp device, we only expect an about 3-fold decrease in particle surface charge, and this effect can be countered by applying higher voltages. In our current design, operating voltages are limited to 0–5 V, but a threefold increase is well within reach, even under high-salinity conditions.

In summary, under the conditions where the highest forces are measured on the particles, electrophoretic interactions contribute approximately 15 fN. In comparison, electroosmotic flow and gravitational interactions contribute only 0.5 fN and 0.05 fN, respectively. These results clearly demonstrate that electrostatic forces dominate and are the primary governing forces in our system. The strength of these electrostatic forces is also influenced by the ionic strength of the solution. Notably, our system can tolerate ionic concentrations up to 300 mM without the need for modifications either on the probe or the device geometry.

## IV. ELECTRIC FIELD CALCULATIONS

We use COMSOL Multiphysics' 'Electric Currents' interface to calculate static electric field distribution in our experimental configuration as the charge relaxation time, $\tau_{\text{water}} = 1.18 \times 10^{-4}$ s, is much smaller than our measurement time in the order of a few tens of seconds. We solve the general form of Ohm's law

$$J = \sigma E + J_e, \tag{6}$$

where $J_e$ is an externally generated current density. Using the static form of the continuity equation,

$$\nabla \cdot J = -\nabla \cdot (\sigma \nabla V - J_e) = 0, \tag{7}$$

the final equation to be solved with the current source becomes

$$Q_j = -\nabla \cdot (\sigma \nabla V - J_e). \tag{8}$$

To calculate the electric field distribution and the electrostatic potential in a water-filled nanochannel, we set the materials and associated electrical conductivity values ($\sigma_{\text{water}} = 5.5 \times 10^{-6}$ S/m, $\sigma_{\text{silica}} = 1 \times 10^{-10}$ S/m, $\sigma_{\text{ITO}} = 1 \times 13.89$ S/m) as well as the desired voltage difference between the ITO terminal (central electrode) and the ITO ground (outer electrodes). We also set the maximum mesh element size to 100 nm around the central electrode, and solve the stationary current conservation equations to obtain electric fields over the system.

In Fig. 2a, we show the change in the electric field distribution as a function of the changing voltage. We observe a linearly increasing electric field in the nanochannel with the applied voltage. In panel b, we show the corresponding electrostatic potentials where we see steeper potential shapes for higher voltages.

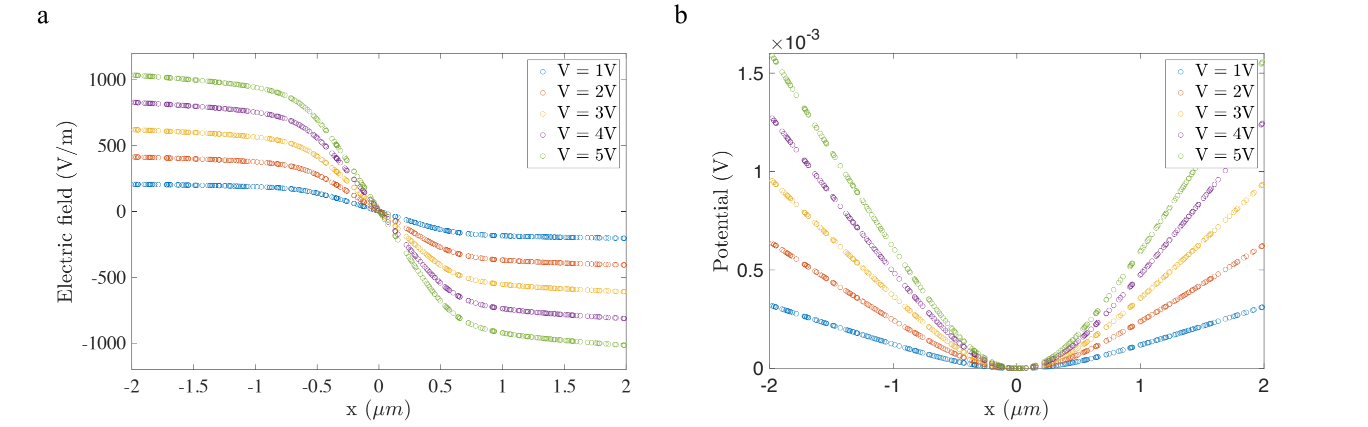


FIG. 2. Calculation of electric field distribution and electrostatic potential. a) The electric field distribution at the mid-plane of the water-filled nanochannel above the central electrode. Higher voltages lead to higher electric fields. b) Corresponding electrostatic potentials are presented. The higher the voltage, the steeper the potential.

Since the electrostatic forces in our system solely depend on the electric charge on the particle ($q$) and the applied electric field, we can estimate $q$ in each measurement. In Fig. 3, we show the distribution of the estimated charges throughout 296 independent measurements by dividing the experimentally estimated forces acting on each particle ($\hat{F}$) by the electric field calculated in COMSOL at that voltage ($E$), $q = \hat{F}/E$. Our estimated charge distribution is in reasonable agreement with reported values in the literature [1, 8].

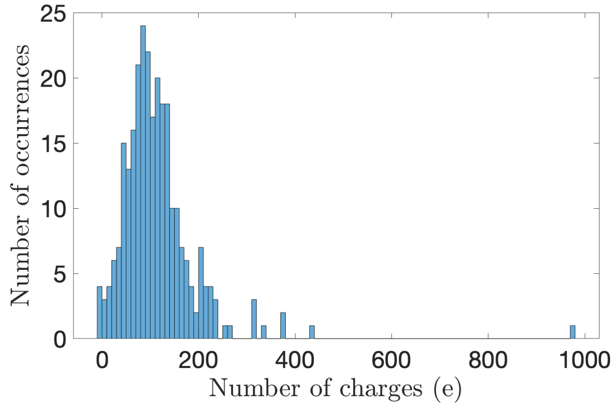


FIG. 3. Distribution of particle charges calculated from experimentally estimated forces and simulated electric fields. The median of the distribution is 101 $e^{-}$.

We also present calculations for other design parameters that allow one to achieve forces at the piconewtons (pN) scale. In Fig. 4a, we display the experimental scheme where $W_w$ is the wire width, $h_w$ is the wire height, $d'$ is the distance between the wire and the channel and $2h$ is the channel height. Here $S$ is the separation of wires and defines the length of the constant force region. It neither significantly affects the shape nor the strength of the potential. We set $S = 35\mu$m as in our original design. $h_w$ does not have any influence on the potential either, and we set it to be thicker ($h_w = 1\mu$m) than our original design for both better electrical and mechanical robustness. $W_w$ affects the characteristic length scale of the system, $d$ (see equation (2) in the main manuscript), namely it determines how far from the trapping center the constant force regime starts. We keep it to be $W_w = 1\mu$m as in our original design.

The two main factors that determine the strength of the electric field inside the channel are the distance between the wire and the channel wall, $d'$, and the channel height, $2h$. Decreasing $h$ causes further squeezing of the electric field, thus creating higher fields. However, increasing $h$ may still be useful when compensated by the probe charge. We set $d'$ to be 50 nm, but it can be further decreased depending on available nanofabrication tools. The electric potential difference between the central and outer electrodes affects the force linearly and can be tuned as needed. We set it to 100 V in our simulations (Fig. 4b) for a fair comparison.

Another evident way of increasing the electrostatic forces on the probe is to increase its charge. For instance, using

larger GNPs with the same surface charge density ($\sigma_{\mathrm{GNP}} = 5 \times 10^3\ e^-/\mu\mathrm{m}^2$), e.g. 2a = 500 nm diameter, in the same configuration, would increase the force by about 40-fold (blue curve in Fig. 4b) compared to a GNP with 2a = 80 nm in size (green curve in Fig. 4b).

We also consider silica beads as conventional probes for optical tweezers. We consider 2a = 800 nm (in a channel $2h = 1$ µm), and 2a = 1 µm (in a channel $2h = 1.2$ µm) silica beads with a surface electron charge density of 700 $e^-/\mu m^2$ [9]. In Fig. 4b, we show that 1µm silica beads experience about 5 pN force.

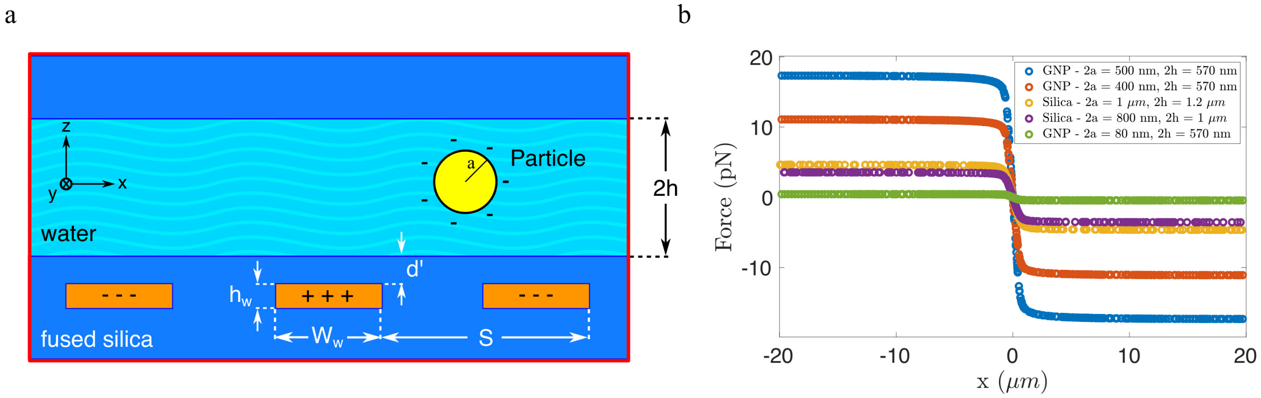


FIG. 4. a) Sketch of an experimental scheme that assigns the dimensions of the setup. b) Forces that gold or silica probes experience under various conditions such as different radii (a) and channel height (2h).

## V. IMAGE ANALYSIS

iSCAT microscopy relies on the interference between a scattered signal (scattering from a particle) with a reference beam (reflected light from glass-water interface) and therefore gives a interferometric point spread function (iPSF) with side interference lobes (See Fig. 5b). In most iSCAT-based experiments [10–12], the particle is tracked by fitting the central minimum or maximum of the iPSF to a Gaussian. However, the radial symmetry of the outer rings can also be used to localize a particle, as achieved by Radial Variance Transform (RVT) [13]. In our analysis, we use RVT because the outer rings of the iPSF are less affected by the scattering from the electrode when the GNP is in its vicinity (See Fig. 5c). We elaborate on this issue below.

Before localizing the particle in each frame, we first remove the static features from each video. Generally, a median background subtraction works well for this, however, if there are any vibrations present, the static features may not be removed completely. Therefore, we use aligned median background subtraction as follows. First, we find the median of all frames and use image registration to align all frames to that median. Then, we calculate the new median of all aligned frames and from each frame, we subtract the new median after aligning it to that frame. Figure 6 shows the raw frame, the result of median background subtraction, and the result of aligned median background subtraction.

After the background is subtracted, we RVT [13], whereby we first define a minimum ($r_{\mathrm{min}} = 20$ pixels) and a maximum ($r_{\mathrm{max}} = 35$ pixels) radius to look for radially symmetric features (shown in red on top of an iPSF in Fig. 7). These values correspond to $3 - 5$ µm. The result of the application of this algorithm to a single frame is shown in Fig. 7 b. The result is then fit to a Gaussian to extract the particle position with sub-pixel precision. The close-up of the result and the Gaussian fit are shown in Fig. 7(c,d). The red cross marks the extracted position of the particle. With this approach, we extract trajectories from the recorded videos. We have over 230 trajectories longer than 10 s with some longer than one minute with a maximum fit error around 0.07 px or less than 10 nm [13].

We have tested the stability of the localization procedure for distorted iPSFs by varying the ring radius in the analysis. We choose $r_{\mathrm{max}} = r_{\mathrm{min}} + 15$ and vary $r_{\mathrm{min}}$ between 0 and 30. The outcome reaches a steady value as the ring size becomes larger because the interference rings are less distorted away from the ITO ribbon. Four exemplary localization cases are presented in Fig. 8.

To quantify the influence of $r_{\mathrm{min}}$ on the localization error, we calculate the Euclidean distance between localizations extracted for subsequent values of $r_{\mathrm{min}}$ for 500 different frames. The results are plotted with thin lines in Fig. 9. The thick line shows the mean values. We see that for small values of $r_{\mathrm{min}}$ the variation in the extracted position is around 100 nm, while for larger values of $r_{\mathrm{min}}$ localizations become a lot more consistent with variations on the order of several nm.

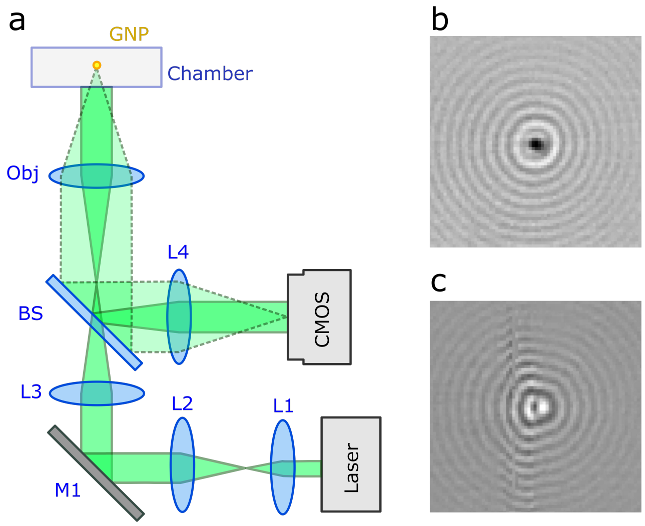


FIG. 5. Interferometric Scattering Microscopy (iSCAT). **(a)** A diagram of the iSCAT realization used in this paper. The light leaves the laser (Laser quantum, GEM 532), goes through a beam expander composed of two lenses (L1:$f = 10$ mm and L2:$f = 200$ mm), then through a wide-field-lens (L3:$f = 400$ mm), and a 50:50 beam splitter. The light is focused on the back focal plane of the microscope objective (Obj: FLUAR, 40X, NA:1.3) which then sends a collimated beam into the chamber. The light reflects from the chamber and gets scattered by the GNP. Both reflected and scattered light return through the objective, get reflected by a beam splitter and are focused by an imaging lens (L4: $f = 300$ mm) on a CMOS camera (Photon focus MV-D1024E-160-CL-12). **(b)** An example of an interferometric PSF (iPSF). The center of the iPSF can be approximated by a Gaussian. **(c)** The experimental iPSF is distorted in the vicinity of the electrode.

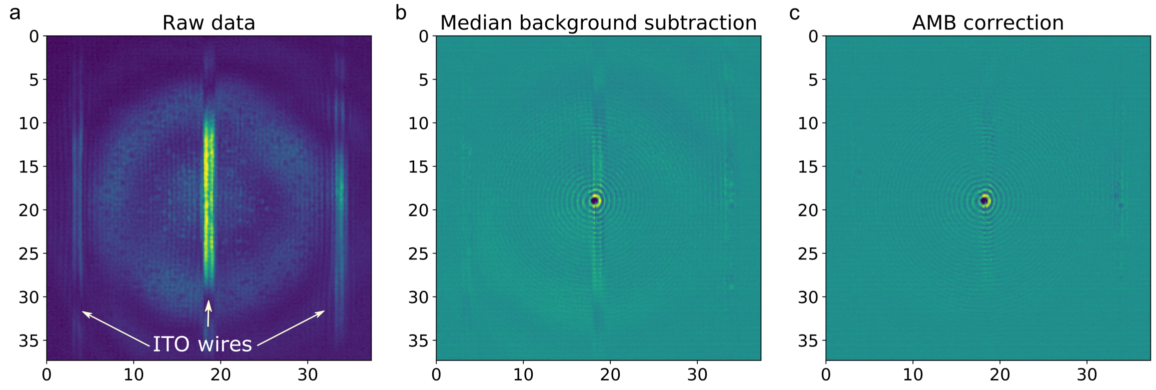


FIG. 6. Comparison of the median and aligned median background subtraction approaches. The axes are in $\mu m$. (a) A typical raw frame in a video. (b) The same image as in (a) after median background subtraction. (c) Same image as in (a) after aligned median background subtraction.

## VI. FORCE ESTIMATION FROM BROWNIAN TRAJECTORIES

### A. Mapping arbitrary potentials

We consider an overdamped Brownian particle under the influence of a smooth external potential $U(x)$. The position, $x(t)$, of this particle is measured at discrete times separated by an interval $\Delta t$. We estimate $U'(x)$ from the experimental time series $(x_0, \ldots, x_n)$, where $x_i = x(i\Delta t)$. For a particle that has achieved its equilibrium distribution, the equilibrium probability density for its position follows the Boltzmann distribution $p(x) = e^{-\beta U(x)}/Z$, where $\beta = 1/k_B T$. This function can be estimated from a histogram of $\{x_i\}$. $U(x)$ is then determined from

$$U(x) = -k_B T \log[Zp(x)]. \tag{9}$$

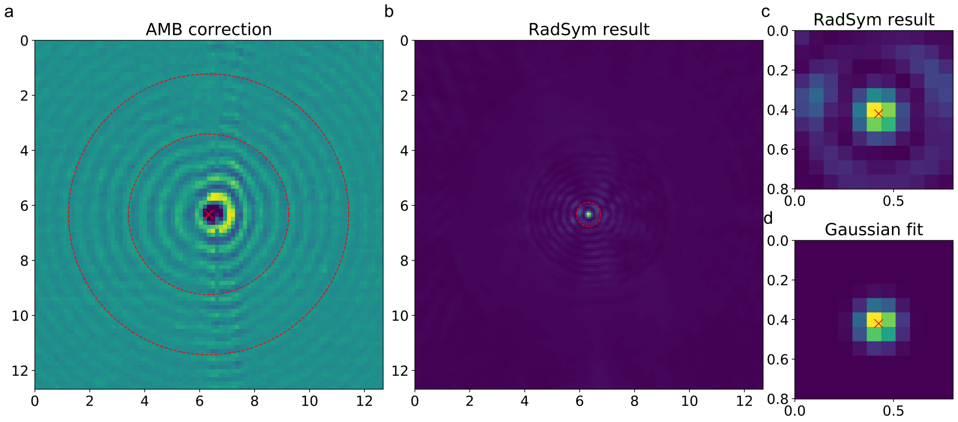


FIG. 7. Radial symmetry algorithm. (a) An experimental iPSF distorted by the presence of the wire. (b) An image generated by the radial symmetry algorithm when applied to the image in panel (a). (c) The close-up of the center of (b). (d) The Gaussian fit to (c).

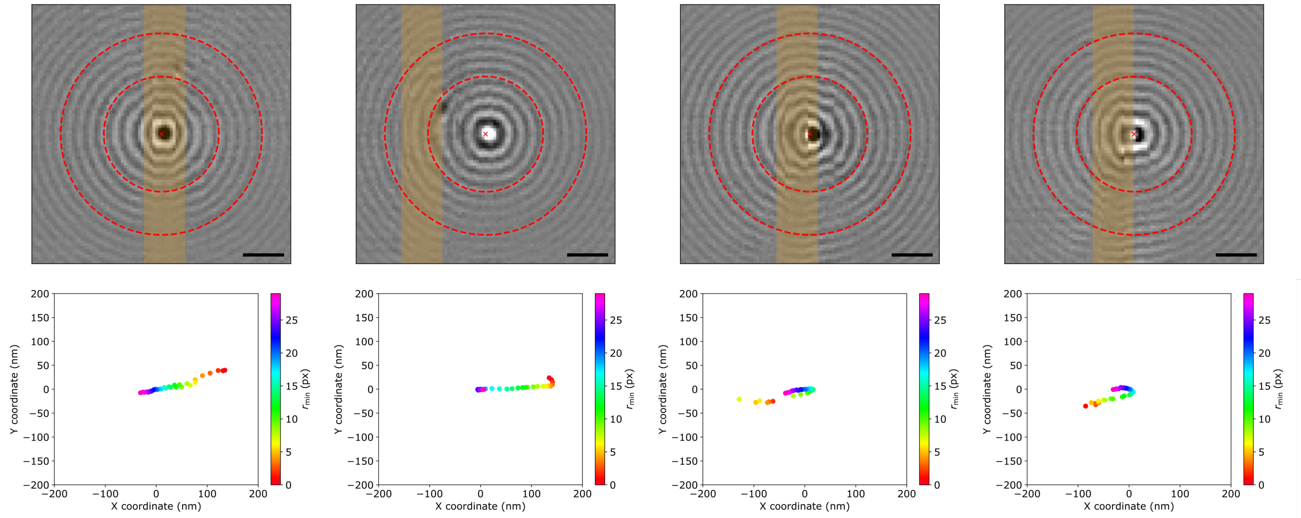


FIG. 8. Effect of choosing $r_{\min}$ in RVT on localization accuracy. The upper panel shows 4 images of a particle diffusing near the embedded central electrode. The rings indicate $r_{\min} = 20$ px and $r_{\max} = 30$ px, which are used in the analysis of the data, while the crosses indicate the extracted positions. The scale bars are 1 µm. The lower panel shows how the localization positions change with the choice of $r_{\min}$, encoded by the color scale. The value of $r_{\max}$ is always 15 pixels larger. The origin of the coordinate system is the localization extracted for $r_{\min} = 20$ px and $r_{\max} = 30$ px.

However, this procedure is not applicable if the experimental trajectories do not reach equilibrium or if the diffusion is nonergodic. We, thus, develop an estimator for $U'(x)$ that applies equally well to equilibrium and out-of-equilibrium trajectories.

Define the step sizes $v_i = x_{i+1} - x_i$ for $i \leq n-1$. $x(t)$ satisfies the following stochastic differential equation

$$dX = -\frac{U'(X)}{\gamma}dt + \sqrt{2D}dW \tag{10}$$

where $\gamma$ is the drag coefficient and $W$ is the standard Wiener process. $v_i$ satisfies

$$\mathbb{E}\left[v_i | x(i\Delta t) = x_i\right] = \mathbb{E}\left[X(t+\Delta t) - x(t) | x(t) = x_i\right] = \frac{U'(x_i)}{\gamma}\Delta t + o(\Delta t) \tag{11}$$

as $\Delta t$ goes to zero. The first equality follows from the Markovian behavior of $x(t)$ and the second from the definition

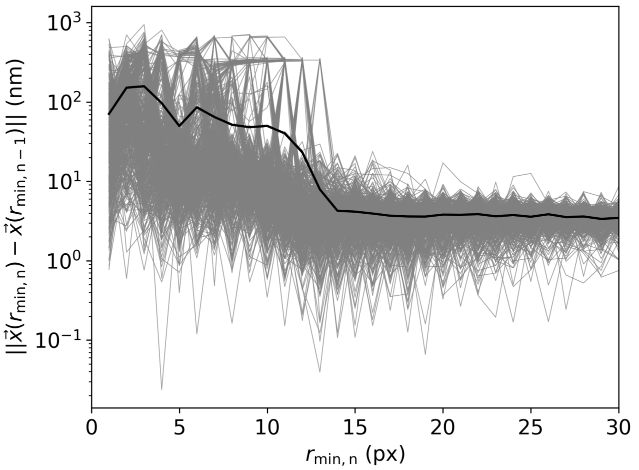


FIG. 9. Euclidean distance between localizations extracted for subsequent values of $r_{\min}$. Thin gray lines are the results from individual frames, while the thick black line denotes the average.

of a diffusion process. This implies

$$\lim_{\Delta t \to 0} \frac{\mathbb{E}\left[v_i | x(i\Delta t) = x_i\right]}{\Delta t} = \frac{U'(x_i)}{\gamma}. \tag{12}$$

Thus, if $\Delta t$ is sufficiently small, the expectation of $v_i$ given $x_i$ is simply $U'(x_i)\Delta t/\gamma$. Intuitively, if the sampling rate is fast enough, $U'$ does not vary significantly over a trajectory that is only $\Delta t$ long, and so the force on the particle during a step is roughly constant. In this limit, if a particle at time $t$ is located at $x_i$, its trajectory over an interval $(t, t + \Delta t)$ is well approximated by a Brownian motion with constant drift $U'(x_i)/\gamma$. We can therefore approximate $U'(x_i)$ as

$$U'(x_i) \approx \frac{\mathbb{E}\left[v_i | x_i\right] \gamma}{\Delta t}. \tag{13}$$

The error in this approximation is on the order of $\sqrt{D\Delta t}\, U''(x_i)$. To estimate $U'(x)$, we require the particle to pass through $x$ a large number of times. Then, $\mathbb{E}\left[v_i | x_i = x\right]$ can be estimated by averaging a population of step sizes that begin at $x$. Since positions are only sampled at discrete intervals, the particle is almost never sampled exactly at $x$. However, we can define a region of width $l$ around $x$ such that $l$ satisfies $l\, U''(x) \ll 1$ at every $x$ within

$$I(x) = (x - l/2\,,\, x + l/2). \tag{14}$$

This means that $U(x)$ is roughly constant within $I(x)$, and so steps that begin within $I(x)$ can be pooled together and averaged to estimate $\mathbb{E}\left[v_i | x_i = x\right]$. From this, we arrive at our estimator for $U'(x)$, which we call $\hat{F}$, given by

$$\hat{F}(x) = \frac{\gamma}{\Delta t} \operatorname{Mean}\{v_i : x_i \in I(x)\}, \tag{15}$$

where the $x_i$ are again the experimental measurements. $\hat{F}(x)$ is not defined if the set $\{v_i : x_i \in I(x)\}$ is empty. Since $U''(x)$ is unknown, we cannot know *a priori* whether $\Delta t$ and $l$ are small enough for this estimator to be valid. This must be determined through convergence tests in $\Delta t$ and $l$ separately.

Besides the errors due to nonzero $l$ and $\Delta t$, there is an additional statistical error due to the estimation of $\mathbb{E}\left[v_i | X(i\Delta t) = x_i\right]$ by sample averaging over $\{v_i : x_i \in I(x)\}$. The standard error in this average can be used to assign a statistical uncertainty to $\hat{F}$. This error falls as $1/\sqrt{N}$, where $N = |\{x_i \in I(x)\}|$ is the number of times the particle is measured within $I(x)$. The error is, therefore, lower for longer trajectories. It should be noted that $N$ is itself a random variable that depends on $x$ because particles are more likely to be sampled near the minima of $U(x)$. This means that $\hat{F}$ has the lowest variance near energy minima (for trapping potentials, $\hat{F}(x)$ is most precise near the trap center and its error increases with $|x|$). The practical consequence of this is the following: when designing an experiment to map a potential using a Brownian particle, there is a trade-off that must be considered when tuning

the diffusion coefficient, $D$. If $D$ is small, the particle only explores a small region near the minima of $U(x)$, and so $U'(x)$ can only be mapped near these minima. If $D$ is increased, the particle explores more of the diffusion space, but $\sqrt{D\Delta t}U''(x)$ will also grow, which worsens the approximation in equation 13. This means that any increase in $D$ must be accompanied by an increase in the sampling rate to suppress the product $D\Delta t$. gamma

### B. Mapping linear potentials

Let us now specialize to the case of linear potentials, which take the form

$$U(x) = F|x| \tag{16}$$

and have a force field $U'(x) = F\,\mathrm{sgn}(x)$. Our problem is to estimate $F$ from a discretely sampled trajectory $(x_0, \dots, x_n)$. If $x_i$ is sufficiently far from zero and $\Delta t$ is sufficiently small, then the step size $v_i$ should be Gaussian with mean $-F\,\mathrm{sgn}(x_i)\Delta t/\gamma$. This is because a trajectory starting far from zero at $x_i$ is unlikely to cross zero and is therefore well approximated by a Brownian motion with constant drift $-F\,\mathrm{sgn}(x_i)/\gamma$. For $x_i$ close to zero, this approximation does not hold. Because $U'(x)$ is not smooth near zero, the approach in section VI A does not apply, and so we will discard steps that start within a threshold $b$ of zero. A suitable estimator for $F$ is then

$$\hat{F} = \frac{\gamma}{\Delta t}\left[\mathrm{Mean}\{-v_i\,\mathrm{sgn}(x_i) : |x_i| > b\}\right]. \tag{17}$$

This is almost equivalent to averaging $|\hat{F}(x)|$ over $|x| > (b + l)$ (where it is defined). Because the appropriate choice of $b$ depends on $D$, $\Delta t$ and also $k$ itself, $b$ is best determined empirically through convergence tests. The statistical uncertainty in $\hat{F}$ can be quantified using the standard error of the set $\{-v_i\,\mathrm{sgn}(x_i) : |x_i| > b\}$.

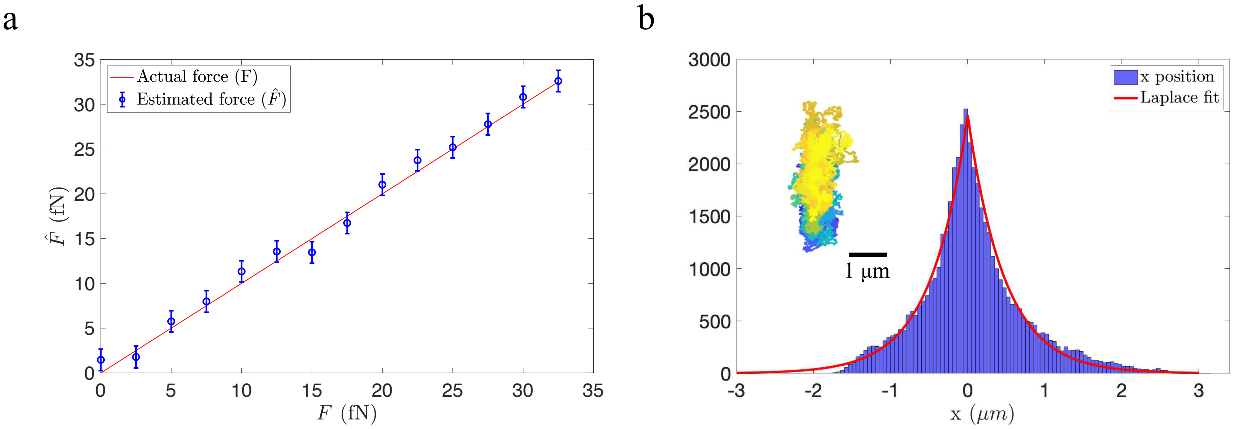


FIG. 10. Validation of the force estimator for a linear potential. a) We estimate $\hat{F}$ from 10 s long simulated trajectories via equation (17) for each data point (blue circles). The results are plotted against the actual force, $F$ (solid red line). Error bars represent the standard deviation in step sizes. b) Laplace fit to the position distribution of a particle whose trajectory is shown in the inset. The estimated force from the fit is, $F = 17.2$ fN, and the prediction of the estimator is $\hat{F} = 19.4$ fN.

This estimator was validated against simulated trajectories in linear potentials of varying magnitude $F$ (Fig. 10a). We also validate $\hat{F}$ on experimental data by using a trajectory that reached stationary (Boltzmann) distribution for a linear potential $U(x) = F|x|$ that is the Laplace distribution

$$p(x) = \frac{\beta}{2} F\,e^{-\beta F|x|}. \tag{18}$$

The position histogram for a sample experimental trajectory is plotted in Fig. 10b (we also show another example in the main text Fig. 3c). The histogram fits well to a Laplace distribution, indicating that the experimental potential is well approximated by a linear potential. The fit was used to estimate $\beta F$ following equation (18). Since $\beta$ is known, this fit provides an independent way of estimating $F$ from the trajectory. The estimate from the histogram is F = 17.2 fN. The estimate given by $\hat{F} = 19.4$ fN is in reasonable agreement.

Lastly, in Fig. 11a, we show the estimated force curves for trajectories recorded on the same particle at different voltages. Here, we estimate linearly increasing forces as the voltage increases. In panel (b), we show the trajectories corresponding to force curves in panel (a).

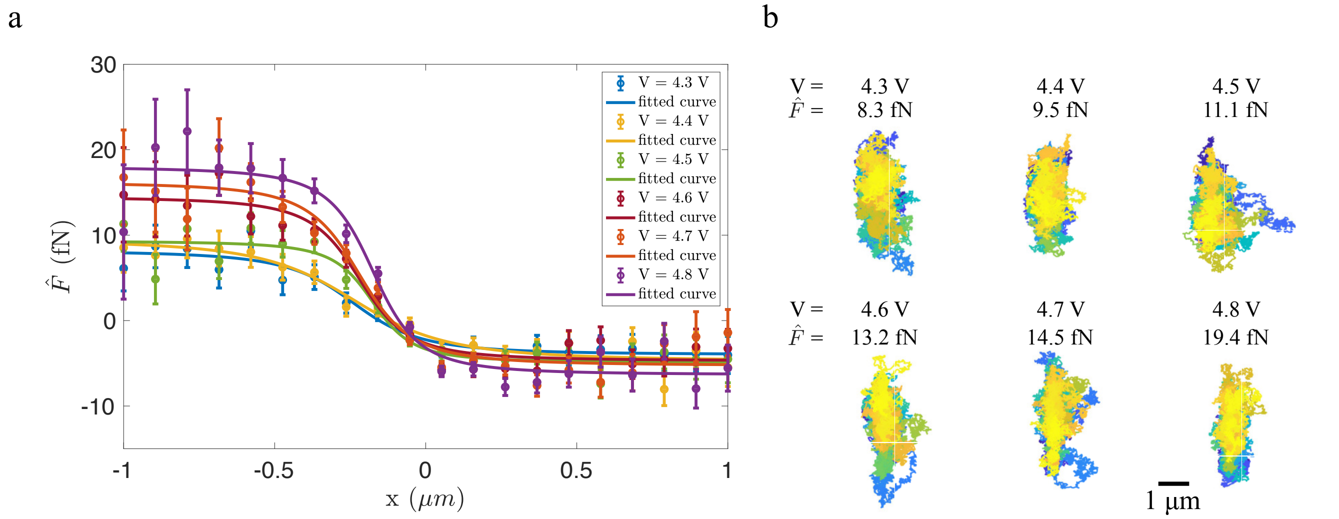


FIG. 11. Force estimation on the same particle and corresponding trajectories. a) Shape of the force fields as a function of distance is given for a range of voltages for the same particle (circles). Solid lines are the fits in the form of $F \times (x + a)/\sqrt{(x+b)^2 + d^2} + c$ where $a$ and $c$ are offsets, and $d$ is the characteristic length scale that is estimated as 238, 333, 141, 172, 183 and 159 nm from 4.3V to 4.8V. b) Corresponding trajectories from panel (a) are presented. Estimated forces ($\hat{F}$) are also shown above each trajectory together with the voltage that the particular trajectory is recorded.

## VII. ANALYTICAL SOLUTION TO MEAN-SQUARED DISPLACEMENT FOR LINEAR POTENTIALS

In this section, we present the analytical formula for the expected behavior asymptotic behavior of MSD curves in the corresponding force field by using the estimated $F_0$, $d$, and $x_0$ values (see equation (3) from the main manuscript) for every single trajectory. The short- and long-time asymptotes are

$$Var(x(t)) \simeq \begin{cases} 2Dt & \text{for t} \to 0 \\ Var_{st}(x) = \dfrac{\int_{-\infty}^{\infty} (x-x_0)^2 e^{-\frac{U(x)}{k_B T}} \, dx}{\int_{-\infty}^{\infty} e^{-\frac{U(x)}{k_B T}} \, dx} & \text{for t} \to \infty \end{cases} \tag{19}$$

where the potential is given by

$$U(x) = F_0 \sqrt{1 + \frac{(x - x_0)^2}{d^2}}. \tag{20}$$


[1] Nasireddin Mojarad and Madhavi Krishnan, "Measuring the size and charge of single nanoscale objects in solution using an electrostatic fluidic trap," Nature Nanotechnology **7** (2012).
[2] M. Krishnan, N. Mojarad, P. Kukura, and V. Sandoghdar, "Geometry-induced electrostatic trapping of nanometric objects in a fluid," Nature **467**, 692–695 (2010).
[3] Jaime Ortega-Arroyo and Philipp Kukura, "Interferometric scattering microscopy (iscat): New frontiers in ultrafast and ultrasensitive optical microscopy," Phys. Chem. Chem. Phys. **14**, 15625–15636 (2012).
[4] Richard W. Taylor and Vahid Sandoghdar, "Interferometric scattering (iscat) microscopy and related techniques," in *Label-Free Super-Resolution Microscopy*, edited by Vasily Astratov (Springer International Publishing, Cham, 2019) pp. 25–65.
[5] Michael A Gerspach, Nassir Mojarad, Deepika Sharma, Thomas Pfohl, and Yasin Ekinci, "Soft electrostatic trapping in nanofluidics," Microsystems & Nanoengineering **3**, 17051 (2017).
[6] Wenjie Wang, Xiaofan Ding, Qing Xu, Jing Wang, Lei Wang, and Xinhui Lou, "Zeta-potential data reliability of gold nanoparticle biomolecular conjugates and its application in sensitive quantification of surface absorbed protein," Colloids and Surfaces B: Biointerfaces **148**, 541–548 (2016).
[7] Ahsan Habib, Masaaki Tabata, and Ying Guang Wu, "Formation of gold nanoparticles by good's buffers," Bulletin of the Chemical Society of Japan **78**, 262–269 (2005).

[8] Carsten Schleh, Manuela Semmler-Behnke, Jens Lipka, Alexander Wenk, Stephanie Hirn, Martin Schäffler, Günter Schmid, Ulrich Simon, and Wolfgang G. Kreyling, "Size and surface charge of gold nanoparticles determine absorption across intestinal barriers and accumulation in secondary target organs after oral administration," Nanotoxicology **6**, 36–46 (2012).

[9] Sven H. Behrens and David G. Grier, "The charge of glass and silica surfaces," The Journal of Chemical Physics **115**, 6716–6721 (2001).

[10] Philipp Kukura, Helge Ewers, Christian Müller, Alois Renn, Ari Helenius, and Vahid Sandoghdar, "High-speed nanoscopic tracking of the position and orientation of a single virus." Nat. Methods **6**, 923–927 (2009).

[11] Chia Lung Hsieh, Susann Spindler, Jens Ehrig, and Vahid Sandoghdar, "Tracking single particles on supported lipid membranes: Multimobility diffusion and nanoscopic confinement," J. Phys. Chem. B **118**, 1545–1554 (2014).

[12] Yazgan Tuna, Ji Tae Kim, Hsuan-Wei Liu, and Vahid Sandoghdar, "Levitated plasmonic nanoantennas in an aqueous environment," ACS Nano **11**, 7674–7678 (2017).

[13] Anna D. Kashkanova, Alexey B. Shkarin, Reza Gholami Mahmoodabadi, Martin Blessing, Yazgan Tuna, André Gemeinhardt, and Vahid Sandoghdar, "Precision single-particle localization using radial variance transform," Optics Express **29**, 11070–11083 (2021).
